# 470. IDSA Chalk Talks as a Catalyst for Enhancing Clinical Teaching and Professional Identity through Medical Education Innovation

**DOI:** 10.1093/ofid/ofaf695.158

**Published:** 2026-01-11

**Authors:** Christian Hendrix, Rachel Bartash, Saira Butt, Michael T Melia, Varun K Phadke, Jennifer O Spicer, Darcy Wooten

**Affiliations:** BJH WashU, St Louis, MO; Montefiore Medical Center, Bronx, NY; Indiana University School of Medicine, Indianapolis, Indiana; Johns Hopkins, Baltimore, Maryland; Emory University, Atlanta, GA; University of Colorado School of Medicine; Washington University in St. Louis, Kirkwood, MO

## Abstract

**Background:**

In 2022, the Infectious Diseases Society of America (IDSA)’s Medical Education Community of Practice (MedEdCOP) launched a novel peer-reviewed platform for ID educators to publish chalk talks —concise, high-yield teaching scripts designed for the clinical learning environment. This open-access library enables educators to disseminate their work and offers ready-to-use teaching materials for others to adopt or adapt. We sought to evaluate the impact of chalk talk authorship on contributors' self-assessed teaching skills, professional identity, and connection to the ID education community.

Demographic Characteristics

**Figure:**
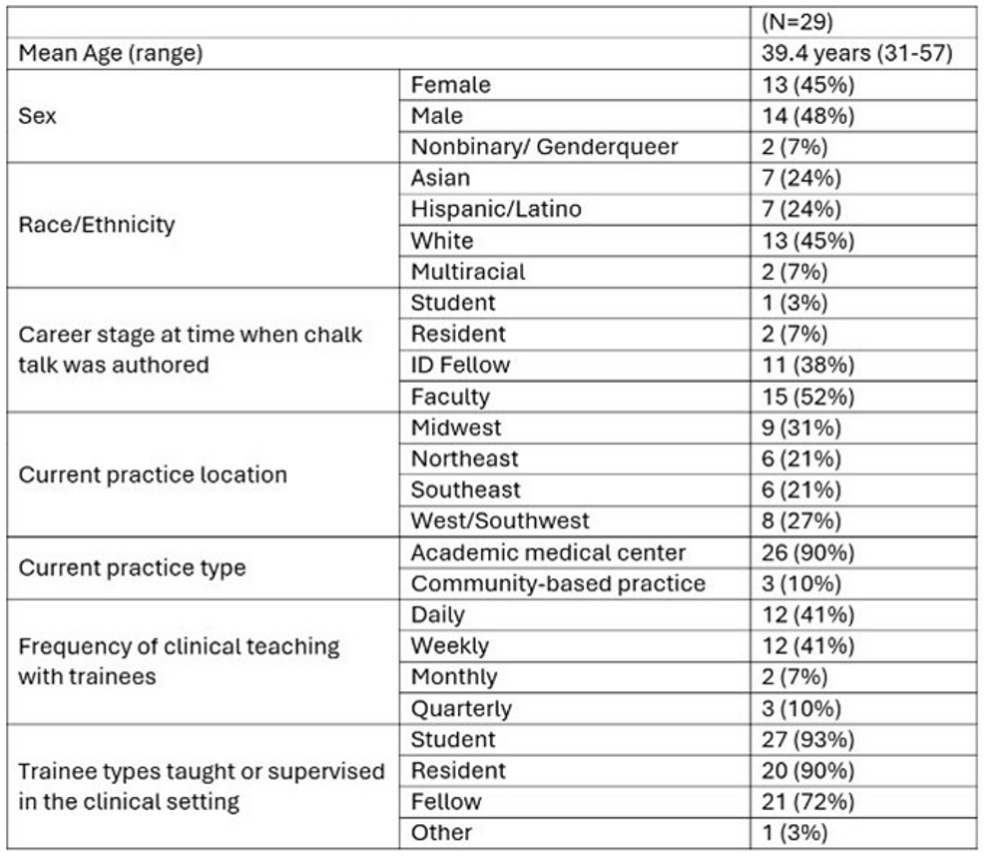
Demographic Characteristics of Chalk Talk Authors

**Figure 1.**
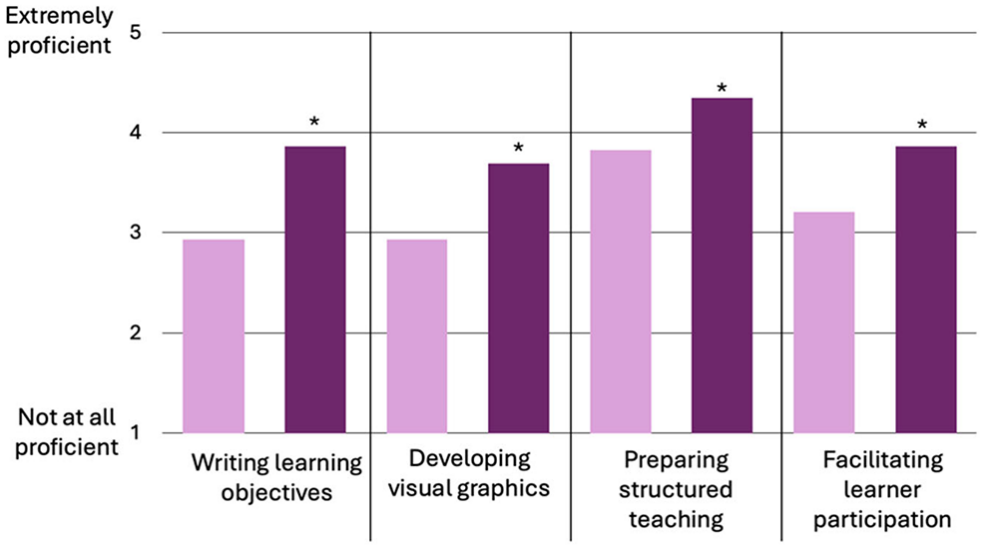
Impact of Chalk Talk Authorship on Clinical Teaching Skills by Self-Assessment Chalk talk authors reported a significant improvement in the proficiency of their clinical teaching skills after publishing an IDSA chalk talk (dark purple) compared to before publishing (light purple). Likert scale for skills rating include: 1- Not at all proficient, 2- Minimally proficient, 3- Somewhat proficient, 4- Very proficient, 5- Extremely proficient. * equates p<0.05. N=29.

**Methods:**

In alignment with best practices in medical education survey design, we developed and distributed an anonymous online survey via Qualtrics LLC. This survey assessed authors' perspectives across the following domains before versus after chalk talk authorship: self-perceived clinical teaching skills, educator identity, value as educators, and sense of connection to IDSA and the MedEdCOP. Paired, two-sided t-tests were used to analyze pre- and post-authorship values. This study was reviewed by the Washington University IRB and classified as exempt.

**Figure 2.**
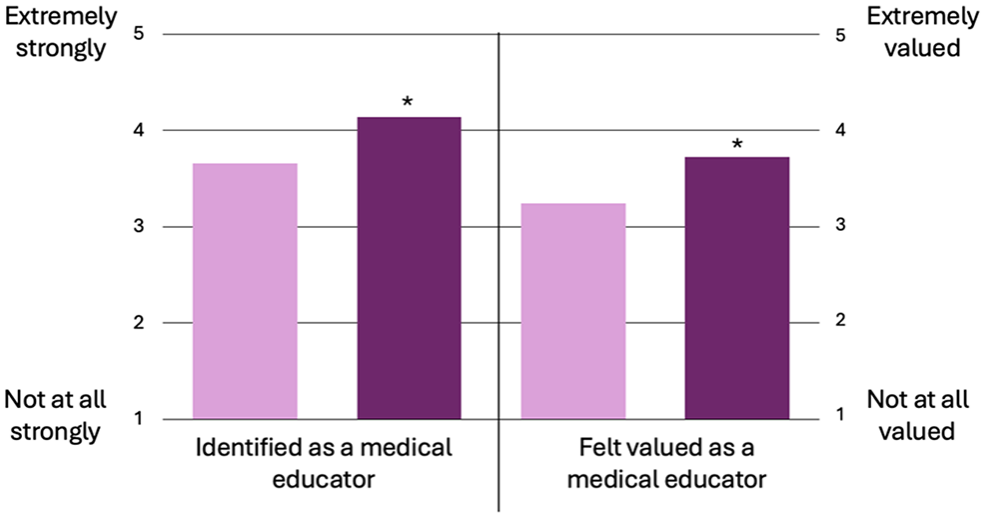
Impact of Chalk Talk Authorship on Professional Identity as a Medical Educator Chalk talk authors reported a significant increase in the degree to which they identified as and felt valued as a medical educator after publishing an IDSA chalk talk (dark purple) compared to before publishing (light purple). Likert scale rating for professional identity includes: 1- Not at all, 2- Minimally, 3- Moderately, 4- Strongly, 5- Very strongly. * equates p<0.05. N=29.

**Results:**

Between 2022 and 2024, 60 chalk talks were published by 41 unique authors through two rounds of peer review. Of these, 29 authors (71%) completed the survey (Table 1). Respondents reported statistically significant improvements in self-assessed clinical teaching skills post-authorship (Figure 1). They also experienced strengthened educator identity and greater perceived value as educators (Figure 2). Additionally, 75% reported an increased post-authorship connection to IDSA and the MedEdCOP. Notably, 73% found the experience helpful in demonstrating scholarly contributions.

**Conclusion:**

Authorship of IDSA chalk talks meaningfully enhanced participants’ self-assessed teaching skills and professional identity, while fostering a sense of value and community. This initiative supports educational innovation and dissemination, contributes to the recruitment and retention of high-quality educators in infectious diseases, and serves as a model for comparable efforts across IDSA and other professional societies.

**Disclosures:**

All Authors: No reported disclosures

